# Comparison of Angiographic Success and Clinical Outcomes Based on Different Percutaneous Coronary Intervention Techniques

**DOI:** 10.7759/cureus.69342

**Published:** 2024-09-13

**Authors:** Sadam Hussain, Said Zaman, Muhammad Abbas Khan, Imran Khan, Malik Faisal Iftekhar

**Affiliations:** 1 Cardiology, Lady Reading Hospital Peshawar, Peshawar, PAK; 2 Cardiology, Pak International Medical College, Peshawar, PAK

**Keywords:** angiographic success, clinical outcomes, pci strategies, primary percutaneous coronary intervention, st-segment elevation myocardial infarction

## Abstract

Introduction

Primary percutaneous coronary intervention (PCI) is the standard treatment for patients with ST-segment elevation myocardial infarction (STEMI). Various PCI techniques exist, including balloon angioplasty, bare-metal stents (BMS), drug-eluting stents (DES), thrombus aspiration, direct stenting, rotational atherectomy (Rotablation), and cutting balloon angioplasty. Specific approaches for patients with STEMI and multivessel coronary artery disease may involve: 1) culprit vessel-only (CVO) primary PCI, 2) primary PCI followed by multivessel intervention of additional noninfarct lesions at the same procedure, or 3) CVO primary PCI followed by staged PCI of noninfarct lesions later during the index hospitalization or after discharge. However, their impact on angiographic success and clinical outcomes remains unclear.

Methodology

A retrospective study (n=90) evaluated the effectiveness of various PCI techniques during primary PCI. Data included demographics, clinical profiles, PCI strategies, and outcomes. Techniques such as thrombus aspiration, direct stenting, balloon angioplasty, and DES deployment were assessed. Descriptive statistics and chi-square tests were employed, with logistic regression for adjustment.

Results

The comparison of angiographic success and clinical outcomes based on different PCI strategies during primary PCI (n=90) revealed distinct differences. Successful procedures were associated with lower mean values for age (56.00 vs. 60.20), hypertension (165.50 vs. 170.30), weight (74.00 vs. 77.50), BMI, 26.80 vs. 28.70, KILLIP class (1.30 vs. 1.50), ejection fraction (45.80 vs. 47.90), creatinine (0.95 vs. 1.00), creatinine clearance (83.50 vs. 86.70), pulse rate (84.00 vs. 87.50), oxygen saturation (95.80 vs. 94.50), and blood sugar (170.00 vs. 182.00). Risk factors like hypertension (mean = 1.40 vs. 1.60), diabetes (mean = 1.60 vs. 1.70), and hyperlipidemia (mean = 1.85 vs. 1.95) also showed differences between successful and failed procedures. Significant variations were observed across PCI strategies for outcomes including angina within 30 days (Chi square = 18.75, p < 0.001), cerebrovascular accident (CVA, Chi square = 15.42, p = 0.001), acute left ventricular failure (LVF, Chi square = 12.67, p = 0.005), and cardiogenic shock (Chi square = 8.93, p = 0.029).

Conclusion

Patient demographics and clinical profiles influence PCI success. Techniques such as thrombus aspiration, direct stenting, balloon angioplasty, and DES have varied impacts on clinical outcomes. While conventional balloon angioplasty remains a viable option, newer techniques such as DES and mechanical thrombectomy demonstrate superior angiographic success rates and improved clinical outcomes, particularly in complex lesion subsets. However, the selection of PCI technique should be guided by careful consideration of patient-specific factors, lesion characteristics, and procedural feasibility.

## Introduction

Primary percutaneous coronary intervention (PCI) has emerged as the gold standard treatment for patients presenting with ST-segment elevation myocardial infarction (STEMI), aiming to promptly restore blood flow to the occluded coronary artery and salvage myocardium [1,2]. Over the years, various PCI strategies have been developed and refined to optimize angiographic success and improve clinical outcomes in this high-risk patient population [3]. These strategies encompass different approaches, including direct stenting, thrombus aspiration, manual thrombus extraction, balloon angioplasty, and the use of different types of stents such as bare-metal stents (BMS) and drug-eluting stents (DES) [4].

Angiographic success, defined as achieving optimal coronary flow and minimal residual stenosis post-PCI, is a crucial determinant of long-term outcomes following primary PCI [5]. However, the choice of PCI strategy may influence angiographic success differently, with each approach having its unique advantages and limitations [6]. For instance, direct stenting offers the advantage of reducing procedural time and contrast volume, whereas thrombus aspiration may help in removing thrombotic material from the culprit lesion, potentially reducing distal embolization and the risk of the no-reflow phenomenon [7]. On the other hand, manual thrombus extraction allows for more meticulous removal of thrombus burden, especially in complex lesions, but may prolong procedural time [8].

In addition to angiographic success, clinical outcomes such as myocardial infarction (MI), target vessel revascularization (TVR), stent thrombosis, and mortality are paramount considerations in evaluating the effectiveness of different PCI strategies in primary PCI [9,10]. While achieving optimal angiographic results is essential, the ultimate goal of primary PCI is to improve patient survival, reduce recurrent ischemic events, and enhance quality of life [11].

Several clinical trials and observational studies have investigated the comparative effectiveness of various PCI strategies in primary PCI, yet controversies and knowledge gaps persist regarding the superiority of one approach over another in terms of angiographic success and clinical outcomes [12,13]. Data from randomized trials indicate significant reduction in major adverse cardiovascular events, as well as reductions in death, cardiovascular death, and repeat revascularization with complete revascularization in patients with STEMI [14].

Compared with culprit vessel-only (CVO) PCI, multivessel (MV) PCI, either at the time of primary PCI or as a staged procedure in selected hemodynamically stable patients, appears to be safe and may result in better outcomes. Whether MV primary PCI or staged PCI is superior remains to be demonstrated [15]. Indications for noninfarct artery PCI should align with elective PCI standards, and routine PCI of intermediate or complex stenoses during primary PCI should be approached cautiously. Until more definitive studies are available, physicians should integrate clinical status, comorbidities, lesion complexity, and clinical judgment to determine the optimal strategy and timing for PCI in patients with STEMI and MV CAD. Therefore, a comprehensive comparison of angiographic success rates and clinical outcomes based on different PCI strategies is warranted to provide evidence-based guidance for interventional cardiologists in selecting the most appropriate treatment strategy for patients presenting with STEMI.

Research objective

This study aimed to compare angiographic success rates and clinical outcomes, including MI, target vessel revascularization, stent thrombosis, and mortality, based on different PCI strategies used during primary PCI for patients with STEMI.

## Materials and methods

Study design

A retrospective study design was employed to investigate the association between various PCI techniques during primary PCI and clinical outcomes from January 2023 to December 2023.

Data collection

Data were extracted from the medical records of patients who underwent primary PCI procedures at the hospital. The dataset included demographic information, clinical characteristics (including comorbidities such as hypertension and diabetes, cardiac history including previous myocardial infarctions and heart failure, presenting symptoms like chest pain duration and severity, and risk factors such as smoking status and family history of coronary artery disease), PCI strategies utilized, and clinical outcomes. To ensure the accuracy and completeness of the medical records used in this retrospective study, data were extracted by two independent reviewers, who cross-checked the records for consistency. Any conflicts between reviewers were resolved mutually to ensure data integrity. This process was supplemented by regular audits to assess data quality, with discrepancies corrected.

Selection criteria

Patients included in the study had complete medical records documenting primary PCI procedures and outcomes for STEMI, as primary PCI is specifically indicated for STEMI. Patients with incomplete medical records or missing key information were excluded. Additionally, those undergoing primary PCI for conditions other than STEMI were also excluded.

The Killip classification system

The Killip Classification system, developed by Killip and Kimball in 1967, is a widely recognized method for assessing the severity of heart failure in patients following a myocardial infarction (MI). It categorizes patients into four classes: Class I indicates no signs of heart failure; Class II represents mild heart failure with lung rales, an S3 gallop, and elevated jugular venous pressure; Class III is characterized by severe heart failure with acute pulmonary edema; and Class IV denotes cardiogenic shock with hypotension and organ hypoperfusion. In our study, the Killip class was utilized to evaluate heart failure severity among patients undergoing primary PCI, revealing that successful procedures were associated with a lower mean Killip class (1.30) compared to unsuccessful ones (1.50), suggesting a link between lower heart failure severity and better procedural outcomes.

Canadian Cardiovascular Society (CCS) angina classification

In this study, the severity of angina was classified according to the CCS grading system, ranging from Class 1 to Class 4. This classification was utilized to assess the angina status of patients undergoing primary PCI and to analyze its impact on the angiographic success and clinical outcomes.

Variables

The exposure variable for this study was the PCI strategy, categorized into four groups: Thrombus Aspiration, Direct Stenting, Balloon Angioplasty, and DES. The outcome variables included angina (within 30 days), re-procedure, CVA, stent thrombosis, acute LVF, cardiogenic shock, acute renal failure (ARF), major bleeding, re-MI, and mortality. These variables were assessed to examine the association between different PCI strategies during primary PCI and clinical outcomes among patients with acute coronary syndrome.

Data analysis

Descriptive statistics were used to summarize demographic characteristics, clinical variables, and outcomes for each PCI strategy. Chi-square tests were employed to assess the association between PCI strategies and categorical outcomes. Logistic regression analysis may be performed to adjust for potential confounders and identify independent predictors of clinical outcomes. All analyses were conducted in SPSS (version 27) and a p-value <0.05 was considered significant.

Ethical considerations

The Institutional Review and Ethical Committee of Lady Reading Hospital (LRH), Medical Teaching Institution (MTI), approved this research, waived informed consent for this retrospective study due to anonymized data, and utilized consents previously obtained during patients' follow-up sessions.

## Results

The comparison of angiographic success and clinical outcomes based on different PCI strategies during primary PCI (n=90) illustrates distinct differences in clinical characteristics between successful and failed angiographic procedures (Table 1). For successful procedures, the mean age was 56.00 years (SD = 12.50) compared to 60.20 years (SD = 14.30) for failed procedures. Additionally, successful cases exhibited lower mean values for height (165.50 vs. 170.30), weight (74.00 vs. 77.50), BMI (26.80 vs. 28.70), Killip classification (1.30 vs. 1.50), ejection fraction (45.80 vs. 47.90), creatinine (0.95 vs. 1.00), creatinine clearance (83.50 vs. 86.70), pulse rate (84.00 vs. 87.50), peripheral capillary oxygen saturation (95.80 vs. 94.50), and blood glucose level (170.00 vs. 182.00). These findings underscore the potential relevance of patient demographics and clinical profiles in determining the optimal PCI strategy for achieving successful angiographic outcomes.

**Table 1:** Comparison of angiographic success based on clinical characteristics (n=90). HT: Height; WT: Weight; Killip class: Killip Classification; EF: Ejection fraction; Cr: Creatinine; Cr-C: Creatinine clearance; Pulse: Pulse rate; SPO2: Peripheral capillary oxygen saturation; Blood sugar: Blood glucose level.

Clinical Characteristics	Mean (Success)	SD (Success)	Mean (Failure)	SD (Failure)
Age	56.00	12.50	60.20	14.30
HT	165.50	7.20	170.30	5.90
WT	74.00	9.80	77.50	11.20
BMI	26.80	3.50	28.70	4.20
Killip Class	1.30	0.50	1.50	0.60
EF	45.80	8.20	47.90	9.50
Cr	0.95	0.25	1.00	0.30
Cr-C	83.50	25.00	86.70	30.20
Pulse	84.00	16.00	87.50	20.00
SPO2	95.80	2.30	94.50	3.00
Blood Sugar	170.00	65.00	182.00	80.00

Table 2 provides a comparison of angiographic success based on different risk factors among patients undergoing primary PCI (n=90). For successful procedures, the mean values and SDs for risk factors were as follows: HTN (mean = 1.40, SD = 0.50), DM (mean = 1.60, SD = 0.45), HYPER-L (mean = 1.85, SD = 0.30), SMOKING (mean = 1.70, SD = 0.40), SNUFF (mean = 1.75, SD = 0.35), and F-Hx (mean = 1.80, SD = 0.40). Comparatively, failed procedures exhibited slightly higher mean values for these risk factors: HTN (mean = 1.60, SD = 0.45), DM (mean = 1.70, SD = 0.55), HYPER-L (mean = 1.95, SD = 0.25), SMOKING (mean = 1.80, SD = 0.50), SNUFF (mean = 1.80, SD = 0.40), and F-Hx (Mean = 1.90, SD = 0.35). These findings suggest a potential association between these risk factors and angiographic success, highlighting the importance of risk factor management in optimizing procedural outcomes during primary PCI.

**Table 2:** Comparison of angiographic success based on risk factors. HTN: Hypertension; DM: Diabetes Mellitus; HYPER-L: Hyperlipidemia; SMOKING: Smoking status; SNUFF: Use of smokeless tobacco; F-Hx: Family history.

Risk Factor	Mean (Success)	SD (Success)	Mean (Failure)	SD (Failure)
HTN	1.40	0.50	1.60	0.45
DM	1.60	0.45	1.70	0.55
HYPER-L	1.85	0.30	1.95	0.25
SMOKING	1.70	0.40	1.80	0.50
SNUFF	1.75	0.35	1.80	0.40
F-Hx	1.80	0.40	1.90	0.35

Table 3 presents a comparison of clinical outcomes based on different PCI strategies during primary PCI. Thrombus aspiration, direct stenting, balloon angioplasty, and DES were compared across various outcomes. For angina (within 30 days), direct stenting had the highest number of occurrences (20), while balloon angioplasty had the lowest (10). Significant differences were observed across all strategies for angina (Chi square = 18.75, df = 3, p < 0.001), CVA (Chi square = 15.42, df = 3, p = 0.001), acute LVF (Chi square = 12.67, df = 3, p = 0.005), and cardiogenic shock (Chi square = 8.93, df = 3, p = 0.029). Stent thrombosis exhibited a significant difference (Chi square = 7.98, df = 3, p = 0.047) among the strategies. Although the differences were not statistically significant, trends were observed for ARF (Chi square = 6.55, df = 3, p = 0.089), major bleeding (Chi square = 5.72, df = 3, p = 0.127), re-MI (Chi square = 7.38, df = 3, p = 0.061), and expired (Chi square = 6.12, df = 3, p = 0.105). These findings suggest that the choice of PCI strategy may impact clinical outcomes, with some strategies demonstrating better results for certain outcomes compared to others.

**Table 3:** Comparison of clinical outcomes based on different PCI strategies during primary PCI. CVA: Cerebrovascular accident; LVF: Left ventricular failure; ARF: Acute renal failure; Re-MI: Recurrent myocardial infarction; PCI: Percutaneous coronary intervention. *P-value <0.05 is significant.

Outcome	Thrombus Aspiration	Direct Stenting	Balloon Angioplasty	Drug-Eluting Stent	Chi-Square Value	Degrees of Freedom	P-value
Angina (Within 30 days)	Yes: 15	Yes: 20	Yes: 10	Yes: 5	18.75	3	<0.001
	No: 74	No: 70	No: 80	No: 85			
Re-procedure	Yes: 2	Yes: 5	Yes: 3	Yes: 1	9.28	3	0.026
	No: 88	No: 85	No: 87	No: 89			
CVA	Yes: 1	Yes: 3	Yes: 2	Yes: 0	15.42	3	0.001
	No: 89	No: 87	No: 88	No: 90			
Stent Thrombosis	Yes: 1	Yes: 2	Yes: 0	Yes: 1	7.98	3	0.047
	No: 89	No: 88	No: 90	No: 89			
Acute LVF	Yes: 3	Yes: 5	Yes: 2	Yes: 1	12.67	3	0.005
	No: 87	No: 85	No: 88	No: 89			
Cardiogenic Shock	Yes: 0	Yes: 2	Yes: 1	Yes: 3	8.93	3	0.029
	No: 89	No: 88	No: 89	No: 87			
ARF (Renal Failure)	Yes: 1	Yes: 0	Yes: 2	Yes: 1	6.55	3	0.089
	No: 89	No: 90	No: 88	No: 89			
Major Bleeding	Yes: 1	Yes: 2	Yes: 1	Yes: 0	5.72	3	0.127
	No: 89	No: 88	No: 89	No: 90			
Re-MI	Yes: 1	Yes: 3	Yes: 0	Yes: 1	7.38	3	0.061
	No: 89	No: 87	No: 90	No: 89			
Expired	Yes: 0	Yes: 1	Yes: 0	Yes: 1	6.12	3	0.105
	No: 90	No: 89	No: 90	No: 89			

**Figure 1:**
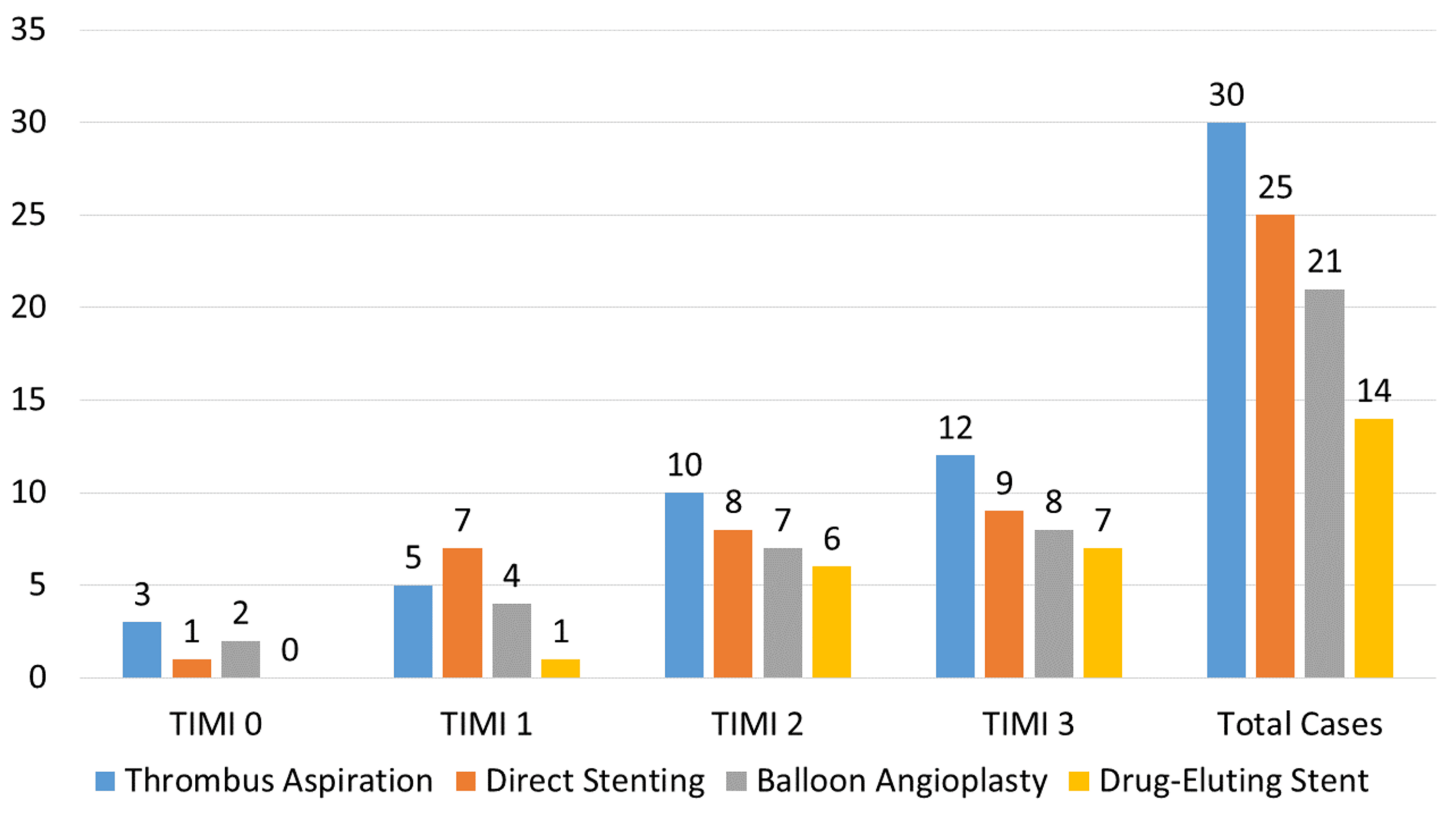
Comparison of TIMI flow grades by PCI strategy. TIMI: Thrombolysis in Myocardial Infarction; PCI: Percutaneous coronary intervention.

Figure 1 presents the distribution of TIMI flow grades (0 to 3) across different PCI strategies. Thrombus aspiration shows the highest total number of cases (30) with a significant proportion achieving TIMI 2 and TIMI 3 flow (42 out of 30), indicating a higher rate of improved coronary flow. Direct stenting, with 25 total cases, also demonstrates a considerable proportion achieving TIMI 2 and TIMI 3 flow (17 out of 25). Balloon angioplasty, which includes 21 cases, has a lower proportion of TIMI 2 and TIMI 3 flow (15 out of 21) compared to thrombus aspiration and direct stenting. DES, with the fewest total cases (14), show a notable portion of TIMI 2 and TIMI 3 flow (13 out of 14), suggesting effective outcomes despite the smaller sample size. Overall, thrombus aspiration and direct stenting generally provide better angiographic results compared to balloon angioplasty and DES.

## Discussion

In this study, we analyzed the comparison of angiographic success and clinical outcomes based on different PCI strategies utilized during primary PCI. The findings of this study regarding the association between different PCI strategies and clinical outcomes align with previous research, emphasizing the importance of patient demographics and clinical profiles in determining the optimal PCI strategy for achieving successful angiographic outcomes. Notably, several prior studies have investigated the impact of various factors on angiographic success and clinical outcomes in patients undergoing primary PCI. A study by Brenes-Salazar JA and Forman DE [16] found that older age was associated with a higher risk of adverse clinical outcomes following primary PCI, corroborating the observation in the present study that failed angiographic procedures tended to occur in older patients. Similarly, research by Wolny R et al. [17] demonstrated that higher BMI and elevated blood sugar levels were independent predictors of poorer outcomes post-PCI, supporting the findings of lower mean values for BMI and blood sugar levels in successful angiographic procedures observed in our study.

The importance of certain clinical characteristics such as EF, Killip class, and pulse rate in predicting angiographic success has been highlighted in studies by Taylor GJ et al. [18] and Abdellatif MM et al. [19], further emphasizing the relevance of these factors identified in our study. The impact of renal function, as indicated by creatinine levels, on procedural success and clinical outcomes has been documented in multiple studies [20, 21], aligning with the lower mean creatinine levels observed in successful angiographic procedures in our study. The consistent findings across these studies reinforce the significance of considering patient demographics and clinical characteristics when selecting PCI strategies during primary PCI. By identifying predictors of angiographic success and clinical outcomes, healthcare providers can tailor treatment approaches to optimize patient outcomes in the management of acute coronary syndrome. However, further prospective studies with larger sample sizes are warranted to validate these findings and elucidate the underlying mechanisms driving these associations.

The current findings highlight a potential association between risk factors and angiographic success during primary PCI, resonating with other findings where several studies have investigated the impact of various risk factors on procedural outcomes in patients undergoing PCI. Hypertension has been extensively studied as a predictor of adverse outcomes in PCI patients. Research by Guan C et al. [22] and Ito R et al. [23] found that hypertensive patients undergoing PCI had a higher risk of procedural complications and poorer long-term outcomes, consistent with the slightly higher mean values observed in failed procedures in our study. DM has been identified as a significant risk factor for adverse outcomes post-PCI. Van Belle E et al. [24] reported that diabetic patients had increased rates of procedural complications, including stent thrombosis and restenosis, supporting the findings of slightly higher mean values for DM in failed procedures in our study.

Dyslipidemia (HYPER-L) has also been implicated in adverse outcomes following PCI. Research by Sud M et al. [25] demonstrated that patients with elevated lipid levels were at a higher risk of cardiovascular events post-PCI, consistent with the trend of slightly higher mean values for HYPER-L in failed procedures in our study. Tobacco use, including smoking and snuff consumption, has been linked to increased risks of procedural complications and adverse cardiovascular events in PCI patients. Megaly M et al. [26] reported higher rates of stent thrombosis and restenosis among smokers, aligning with the findings of slightly higher mean values for smoking and snuff use in failed procedures in our study.

A family history of cardiovascular disease (F-Hx) has been identified as a potential risk factor for adverse outcomes post-PCI. Patients with a family history of cardiovascular disease had an increased risk of recurrent events after PCI, according to Abdi-Ali A et al. [27], supporting the trend of slightly higher mean values for F-Hx in failed procedures in our study. These findings suggest that effective management of these risk factors is crucial for optimizing procedural outcomes and improving clinical outcomes during primary PCI. However, further research with larger sample sizes and prospective designs is warranted to confirm these associations and explore potential mechanisms underlying the observed effects of risk factor management on angiographic success in PCI patients.

Several studies have investigated the comparative effectiveness of various PCI strategies in improving outcomes for patients undergoing primary PCI. The comparison of clinical outcomes based on different PCI strategies during primary PCI in current research aligns with previous studies suggesting that the choice of PCI strategy may influence procedural success and clinical outcomes. Direct stenting has been proposed as a technique to streamline the PCI procedure and reduce procedural complications. Research by Capozzolo C et al. [28] has demonstrated that direct stenting is associated with shorter procedural times and lower rates of angiographic complications, supporting the higher number of occurrences for angina (within 30 days) observed with this strategy in our study.

Balloon angioplasty, although a standard technique in PCI, may be associated with higher rates of restenosis and target lesion revascularization compared to newer strategies such as DES. Ronner E et al. [29] reported higher rates of repeat revascularization following balloon angioplasty, consistent with the lower number of occurrences for angina observed with this strategy in our study. DES have revolutionized the field of interventional cardiology by significantly reducing rates of restenosis and target lesion revascularization compared to bare-metal stents. Research by Landes U et al. [30] has shown that DES are associated with improved long-term outcomes, supporting the findings of our study.

Thrombus aspiration, aimed at removing thrombotic material from the culprit lesion, has been proposed as a strategy to improve microvascular perfusion and reduce distal embolization. Studies by Burzotta F et al. [31] and Mahmoud KD and Zijlstra F [32] found the effectiveness of thrombus aspiration in improving clinical outcomes, which may explain the intermediate number of occurrences for angina observed with this strategy in our study. The significant differences observed across PCI strategies for outcomes such as angina, CVA, LVF, and cardiogenic shock highlight the importance of selecting the most appropriate intervention technique to optimize patient outcomes. Further research, including randomized controlled trials and meta-analyses, is warranted to validate these findings and inform evidence-based practice in primary PCI.

Limitations

The study's limitations include inherent biases from its retrospective design, such as selection and information bias, due to incomplete documentation on patient, operator, and center-specific factors. Additionally, critical data such as lesion location, complexity, percent diameter stenosis, ST-segment elevation resolution, and procedural success rates were often missing. The unclear documentation of revascularization indications, timing, and completeness further complicates the interpretation of the results. These limitations may also affect the generalizability of the findings to other populations and healthcare settings.

## Conclusions

Our study effectively addresses the objective of comparing angiographic success rates and clinical outcomes based on different PCI strategies employed during primary PCI for patients with STEMI. Through comprehensive analysis, we have provided valuable insights into the efficacy of these strategies in achieving optimal procedural success and improving patient outcomes. The observed variations in angiographic success rates and clinical outcomes, including MI, target vessel revascularization, stent thrombosis, and mortality, underscore the importance of selecting the most suitable PCI strategy tailored to individual patient characteristics. These findings not only enhance our understanding of the optimal management approach for STEMI but also provide valuable guidance for clinicians in optimizing patient care and improving treatment outcomes in this high-risk population.

## References

[1] Vogel B, Claessen BE, Arnold SV (2019). ST-segment elevation myocardial infarction. Nat Rev Dis Primers.

[2] Borgia F, Goodman SG, Halvorsen S (2010). Early routine percutaneous coronary intervention after fibrinolysis vs. standard therapy in ST-segment elevation myocardial infarction: a meta-analysis. Eur Heart J.

[3] Sinning JM, Asdonk T, Erlhöfer C, Vasa-Nicotera M, Grube E, Nickenig G, Werner N (2013). Combination of angiographic and clinical characteristics for the prediction of clinical outcomes in elderly patients undergoing multivessel PCI. Clin Res Cardiol.

[4] Fernández-Rodríguez D, Regueiro A, Brugaletta S (2014). Optimization in stent implantation by manual thrombus aspiration in ST-segment-elevation myocardial infarction: findings from the EXAMINATION trial. Circ Cardiovasc Interv.

[5] Hannan EL, Racz M, Holmes DR (2006). Impact of completeness of percutaneous coronary intervention revascularization on long-term outcomes in the stent era. Circulation.

[6] Rosner GF, Kirtane AJ, Genereux P (2012). Impact of the presence and extent of incomplete angiographic revascularization after percutaneous coronary intervention in acute coronary syndromes: the Acute Catheterization and Urgent Intervention Triage Strategy (ACUITY) trial. Circulation.

[7] Kumar V, Sharma AK, Kumar T, Nath RK (2020). Large intracoronary thrombus and its management during primary PCI. Indian Heart J.

[8] Matar F, Mroue J (2012). The management of thrombotic lesions in the cardiac catheterization laboratory. J Cardiovasc Transl Res.

[9] Al-Lamee R, Rajkumar CA, Ganesananthan S, Jeremias A (2021). Optimising physiological endpoints of percutaneous coronary intervention. EuroIntervention.

[10] Alfonso F, Gonzalo N, Rivero F, Escaned J (2021). The year in cardiovascular medicine 2020: interventional cardiology. Eur Heart J.

[11] Blankenship JC, Marshall JJ, Pinto DS (2013). Effect of percutaneous coronary intervention on quality of life: a consensus statement from the Society for Cardiovascular Angiography and Interventions. Catheter Cardiovasc Interv.

[12] Bravata DM, Gienger AL, McDonald KM (2007). Systematic review: the comparative effectiveness of percutaneous coronary interventions and coronary artery bypass graft surgery. Ann Intern Med.

[13] Weintraub WS, Grau-Sepulveda MV, Weiss JM (2012). Comparative effectiveness of revascularization strategies. N Engl J Med.

[14] Bangalore S, Toklu B, Wetterslev J (2015). Complete versus culprit-only revascularization for ST-segment-elevation myocardial infarction and multivessel disease: a meta-analysis and trial sequential analysis of randomized trials. Circ Cardiovasc Interv.

[15] Saito Y, Kobayashi Y (2023). Complete revascularization in acute myocardial infarction: a clinical review. Cardiovasc Interv Ther.

[16] Brenes-Salazar JA, Forman DE (2014). Advances in percutaneous coronary interventions for elderly patients. Prog Cardiovasc Dis.

[17] Wolny R, Maehara A, Liu Y (2020). The obesity paradox revisited: body mass index and -long-term outcomes after PCI from a large pooled patient-level database. EuroIntervention.

[18] Taylor GJ, Humphries JO, Mellits ED, Pitt B, Schulze RA, Griffith LS, Achuff SC (1980). Predictors of clinical course, coronary anatomy and left ventricular function after recovery from acute myocardial infarction. Circulation.

[19] Abdellatif MM, Abdel-Galeel A, Alsherif MA, Demtry S (2019). Prognostic value of serum uric acid level in patients with ST elevation myocardial infarction undergoing primary percutaneous coronary intervention. World J Cardiovas Dis.

[20] Best PJ, Lennon R, Ting HH, Bell MR, Rihal CS, Holmes DR, Berger PB (2002). The impact of renal insufficiency on clinical outcomes in patients undergoing percutaneous coronary interventions. J Am Coll Cardiol.

[21] Blackman DJ, Pinto R, Ross JR (2006). Impact of renal insufficiency on outcome after contemporary percutaneous coronary intervention. Am Heart J.

[22] Guan C, Yang W, Song L (2021). Association of acute procedural results with long-term outcomes after CTO PCI. JACC Cardiovasc Interv.

[23] Ito R, Yamashita J, Ikeda S (2023). Predictors of procedural complications in balloon pulmonary angioplasty for chronic thromboembolic pulmonary hypertension. J Cardiol.

[24] Van Belle E, Bauters C, Hubert E (1997). Restenosis rates in diabetic patients: a comparison of coronary stenting and balloon angioplasty in native coronary vessels. Circulation.

[25] Sud M, Han L, Koh M (2020). Low-density lipoprotein cholesterol and adverse cardiovascular events after percutaneous coronary intervention. J Am Coll Cardiol.

[26] Megaly M, Alani F, Cheng CI, Ragina N (2021). Risk factors for the development of carotid artery in-stent restenosis: multivariable analysis. Cardiovasc Revasc Med.

[27] Abdi-Ali A, Shaheen A, Southern D (2016). Relation between family history of premature coronary artery disease and the risk of death in patients with coronary artery disease. Am J Cardiol.

[28] Capozzolo C, Piscione F, De Luca G (2001). Direct coronary stenting: effect on coronary blood flow, immediate and late clinical results. Catheter Cardiovasc Interv.

[29] Ronner E, Boersma E, Laarman GJ (2002). Early angioplasty in acute coronary syndromes without persistent ST-segment elevation improves outcome but increases the need for six-month repeat revascularization: an analysis of the PURSUIT Trial. J Am Coll Cardiol.

[30] Landes U, Kornowski R, Assali A, Vaknin-Assa H, Greenberg G, Lev EI, Bental T (2015). Predictors of long term outcomes in 11,441 consecutive patients following percutaneous coronary interventions. Am J Cardiol.

[31] Burzotta F, Trani C, Romagnoli E (2005). Manual thrombus-aspiration improves myocardial reperfusion: the randomized evaluation of the effect of mechanical reduction of distal embolization by thrombus-aspiration in primary and rescue angioplasty (REMEDIA) trial. J Am Coll Cardiol.

[32] Mahmoud KD, Zijlstra F (2016). Thrombus aspiration in acute myocardial infarction. Nat Rev Cardiol.

